# Spontaneous Bladder Rupture after Normal Vaginal Delivery: Description of a Rare Complication and Systematic Review of the Literature

**DOI:** 10.3390/diagnostics11101885

**Published:** 2021-10-13

**Authors:** Guglielmo Stabile, Francesco Cracco, Davide De Santo, Giulia Zinicola, Federico Romano, Nicolò De Manzini, Serena Scomersi, Giuseppe Ricci

**Affiliations:** 1 Institute for Maternal and Child Health, IRCCS “Burlo Garofolo”, Via dell’Istria 65/1, 34137 Trieste, Italy; davide.desanto@burlo.trieste.it (D.D.S.); federico.romano@burlo.trieste.it (F.R.); giuseppe.ricci@burlo.trieste.it (G.R.); 2 UCO Clinica Ostetrica e Ginecologica, Department of Medicine, Surgery and Health Sciences, University of Trieste, 34137 Trieste, Italy; francesco.cracco@burlo.trieste.it (F.C.); giulia.zinicola@burlo.trieste.it (G.Z.); 3 SC (UCO) Clinica Chirurgica, Department of Medicine, Surgery and Health Sciences, University of Trieste, Strada di Fiume 447, 34149 Trieste, Italy; nicolo.demanzini@asugi.sanita.fvg.it (N.D.M.); serena.scomersi@asugi.sanita.fvg.it (S.S.)

**Keywords:** bladder rupture, spontaneous, vaginal delivery, systematic review, laparoscopy

## Abstract

Objective: To identify the possible causes of spontaneous bladder rupture after normal vaginal delivery and to propose a diagnostic and therapeutic algorithm. Material and Methods: MEDLINE (PubMed), Web of Science and Scopus databases were searched up to August 2020. Manuscripts considered were published from 1990 and only English articles were included. The research strategy adopted included the following terms: (bladder rupture) AND (spontaneous) AND (delivery). 103 studies were identified. Duplicates were found through an independent manual screening. Subsequently, two authors independently screened the full text of articles and excluded those not pertinent to the topic. Discrepancies were resolved by consensus. Finally, thirteen studies were included. Results: PRISMA guidelines were followed. For each study, fetal weight, catheterization during labor, parity, maternal age, occurrence time, previous abdominal or pelvic surgery, symptoms complained of, diagnostic methods, and treatment were considered. Median age was 26.0 (range 20–34 years); median presentation time was 3.0 days after delivery (range 1–20 days); and median newborn weight was 3227.0 g (range 2685–3600 g). Catheterization during labor was reported only in four of the thirteen cases (30.8%) identified. The symptoms most frequently complained of were abdominal pain and distension, fever, oliguria, haematuria and vomiting. Instrumental diagnosis was performed using X-rays in five cases and computerized tomography in six cases. Ultrasound was chosen in five cases as a first diagnostic tool. In two cases, cystography was performed. Treatment was always laparotomic repair of the visceral defect. Conclusion: Abdominal pain, increased creatinine and other signs of kidney failure on blood tests should lead to suspicion of this complication. Cystourethrography is regarded as a procedure of choice, but a first ultrasound approach is recommended. The main factor for the therapeutic choice is the intraperitoneal or extraperitoneal rupture of the bladder. Classical management for intraperitoneal rupture of the bladder is surgical repair and urinary rest.

## 1. Introduction

Spontaneous bladder rupture during labor or postpartum is an extremely rare condition. Increased visceral pressure, weakening of the bladder, and vesical catheterization performed during labor are predisposing factors [1]. An increased intraperitoneal pressure intrapartum and postpartum has been reported to cause bladder rupture. Signs and onset symptoms typically consist of ascites and acute abdominal pain. Irritation due to urine following intraperitoneal rupture may result in peritonitis or sepsis [2]. Patients may complain of suprapubic pain, anuria, and hematuria; in rare cases, intraperitoneal bladder rupture may not be associated with abdominal pain and urine may be passed without any symptoms, and so the diagnosis of intraperitoneal rupture may be difficult in these situations. The contextual finding of elevated serum urea and creatinine should raise the possibility of bladder rupture [3]. Some reports have noticed pseudo-renal failure because of creatinine diffusion into the circulation [4].

Surgery is crucial for the resolution of the clinical picture and consists of urine removal from the peritoneal cavity and closing of the rupture [5].

This complication represents a surgical emergency, and a rapid diagnosis represents a challenge for gynecologists.

We performed a systematic review of the literature to identify the possible causes of spontaneous bladder rupture after normal vaginal delivery and to propose a diagnostic and therapeutic algorithm. We report a case of a bladder rupture the second day after a spontaneous vaginal delivery, managed in our department.

## 2. Case

**Figure 1:**
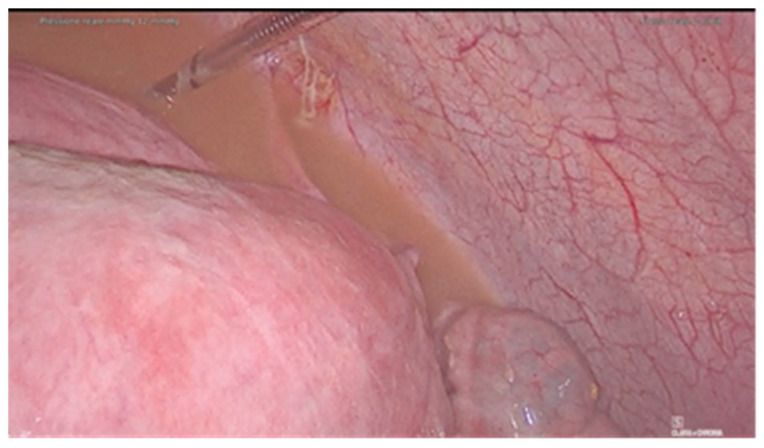
Uroperitoneum.

**Figure 2:**
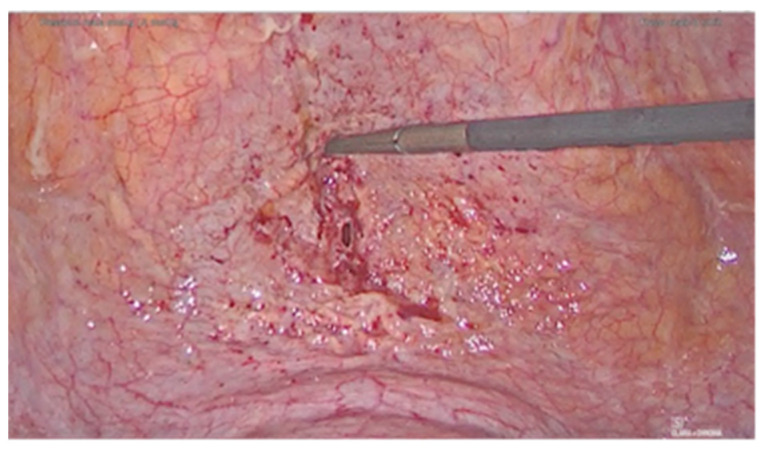
Bladder dome injury.

**Figure 3:**
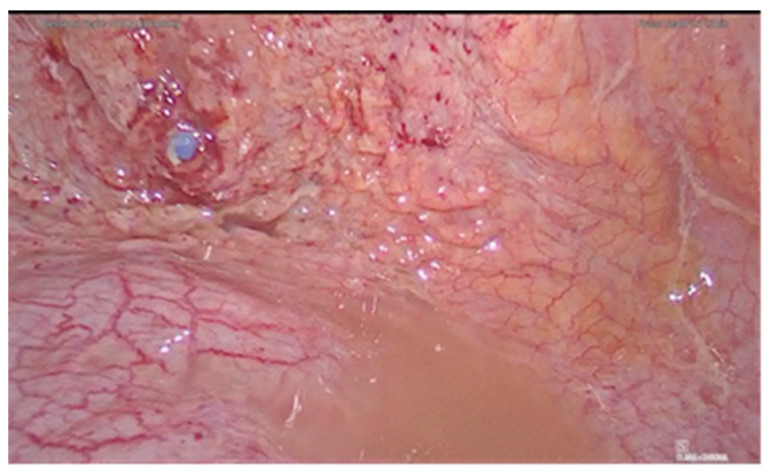
Bladder dome injury, view with vesical catheter.

**Figure 4:**
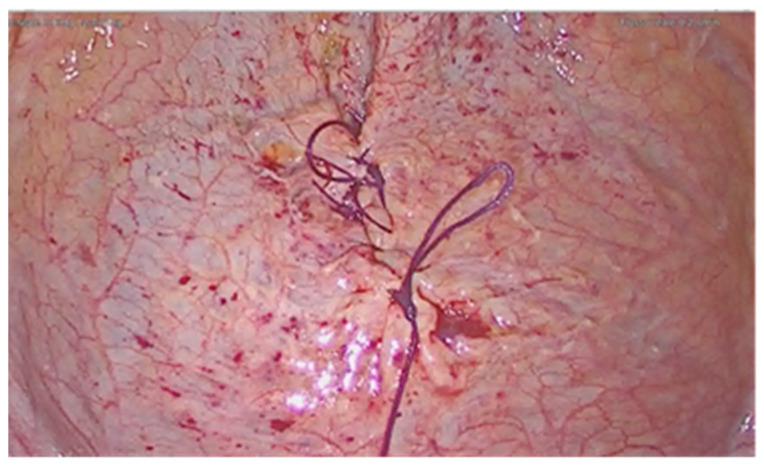
Suture of the vesical injury.

A 36-year-old woman was diagnosed with a bladder rupture on the second day after a spontaneous vaginal delivery which was performed successfully. The patient reported sudden onset of acute abdominal pain with no apparent cause. She had an uneventful pregnancy without any genitourinary problems. Her medical history included no major pathologies and no previous surgical procedures. During labor, she was unable to empty her bladder spontaneously, and so a urinary catheter was used. The day after delivery, diuresis and peristalsis were regular. The patient reported any other symptoms, such as nausea and vomiting. Physical examination revealed a generalized abdominal tenderness. An office trans-abdominal ultrasound showed a copious abdominal effusion while blood chemistry tests reflected a septic state with high creatinine levels. A broad-spectrum antibiotic therapy was issued immediately, with 4.5 g of piperacillin-tazobactam to be taken four times daily. A CT-scan was urgently performed and confirmed the presence of a massive free abdominal effusion, showing the presence of intestinal loops with thickened walls. The bladder appeared intact. An urgent exploratory laparoscopy was decided on and about 2 L of fluid was suctioned and a diagnosis of uroperitoneum was made (Figure 1). An intraperitoneal rupture in the dome of the bladder was highlighted (Figure 2 and Figure 3). The injury was about 0.5 cm and repaired with continuous suture in two layers using Vicryl 3.0 (Figure 4). The patient was catheterized for the first 7 days after surgery. In the second week after surgery, the diuresis and the post-micturition residue were carefully controlled; an ultrasound check was made every three hours and if the urinary retention exceeded 500 cc, the patient was subjected to catheterization. Therapy with tamsulosin improved spontaneous diuresis, avoiding the need for multiple catheterizations. Eleven days after surgery a cystourethrography was performed with normal results. However, the patient developed mixed obstructive-irritative urinary symptoms with a post inflammatory state, as confirmed by urologists. At the 3-month follow-up, the patient was in good clinical condition and asymptomatic.

## 3. Materials and Methods

This research was approved by our Institutional Review Board (RC 08/2020).

MEDLINE (PubMed), Web of Science and Scopus databases were searched up to August 2020. The manuscripts considered were published from 1990 up to August 2020. Only articles in English were included in the search. The research strategy adopted included different combinations of the following terms: (bladder rupture) AND (spontaneous) AND (delivery).

For the selection of the papers, we included articles that focused on spontaneous bladder rupture during or after a normal vaginal delivery. We examined in our review the age of patients, their obstetrical history, previous surgical procedures, catheterization during labor, symptoms, the type of diagnosis and outcome.

We excluded from the review studies concerned with cases of bladder rupture that occurred after an operative vaginal delivery, during cesarean section delivery, in women with a history of previous cesarean section delivery or pelvic surgery or previous genitourinary mutilation. Articles not relevant to the topic were also excluded.

**Figure 5:**
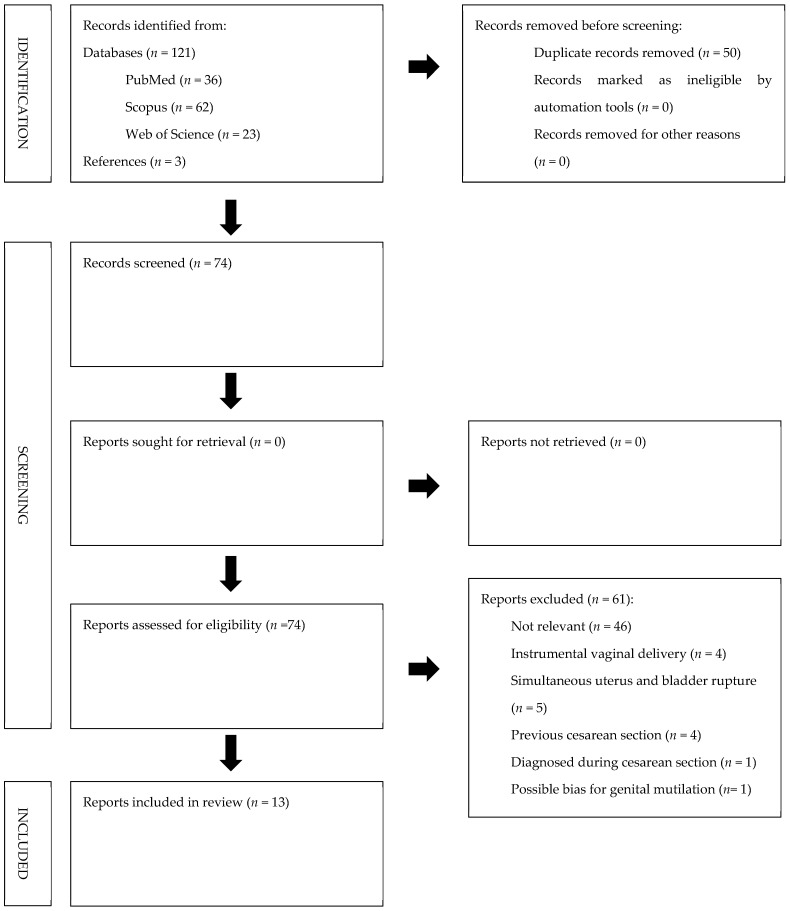
PRISMA flow diagram.

All studies identified were examined for year, citation, title, authors, abstract and their full texts. Duplicates were identified through manual screening performed by one researcher and then removed. PRISMA guidelines were followed [6]. The PRISMA flow diagram of the selection process is provided in Figure 5. The systematic review was not submitted to Prospero [7] as only a limited number of case reports were found in the literature. For the eligibility process, two authors independently screened the title and abstracts of all non-duplicated papers and excluded those not pertinent to the topic. The same two authors independently reviewed the full text of papers that passed the first screening and identified those to be included in the review. Discrepancies were resolved by consensus.

Two manuscripts were detected through the references of the works that had been identified with the research on PubMed and Scopus.

Two researchers performed data extraction using a predefined form including the following data: author, month and year.

Due to the rarity of this pathology, the studies included are all case reports. For this reason, we present the data in a descriptive manner. The inclusion of only case reports in this review presents a risk of bias. The methodological quality of the included studies was assessed using the JBI Critical Appraisal Checklist for case reports (Table 1 and Table A1).

## 4. Results

We identified 106 manuscripts. Records identified through databases searching were 103 (*n* = 36 from MEDLINE; *n* = 62 from Scopus; *n* = 5 from Web of Science). Three manuscripts were detected through the references of the works that had been identified with the search of MEDLINE, Web of Science and Scopus. Records excluded for selection criteria and duplicates were *n* = 91. Two other manuscripts were excluded as they showed simultaneous postpartum rupture of the uterus and bladder. We included in our review a total of thirteen cases at the end of the screening process (see Table 2).

In our analysis the median age of women affected by the spontaneous bladder rupture after delivery is 26.0 (range 20–34 years). Referring to the median value, the diagnosis occurs 3.0 days after delivery, with the latest diagnosis occurring at 20 days after delivery and the earliest at 12 h. Fetal weight was not found to be a determining factor for bladder rupture. In the thirteen cases identified, the fetal weight is very variable, ranging from a maximum of 3600 g to a minimum of 2685, with the median weight of the newborn being 3227.0 g. Even parity was not found to be a major risk factor for rupture of the bladder. Of the thirteen cases identified, eight occurred in the first pregnancy (61.5%), and three in the second (23.1%) while two were not known (15.4%). Regarding our patient, it was her first pregnancy. Catheterization during labor is reported only in four of the thirteen cases identified (30.8%).

The symptoms most frequently manifested by patients were abdominal pain (53.9%), abdominal distension (38.5%), fever (15.4%), oliguria (38.5%), haematuria (15.4%) and vomiting (15.4%).

**Figure 6:**
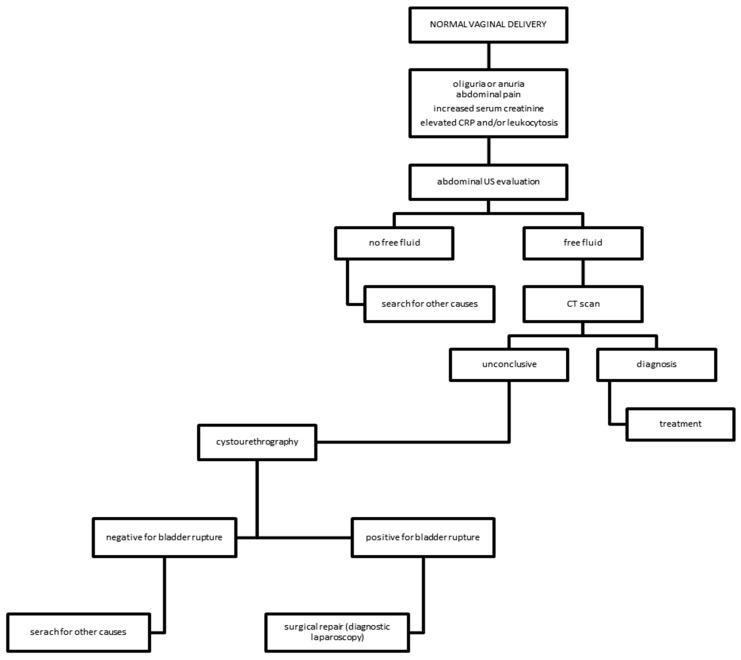
Diagnostic algorithm for bladder rupture following normal vaginal delivery.

The instrumental diagnosis was made with an X-ray of the abdomen (five cases, 38.5%), computerized tomography, and abdominal ultrasound (US). Abdominal X-ray was used in five cases; computerized tomography was used solely in two cases, one time after abdominal X-ray and three times after ultrasound evaluation. US was used in five cases as a first diagnostic tool. In two cases, a specific diagnostic tool for the urinary tract was used, such as cystography. On the basis of the results of our review, we suggest a diagnostic algorithm (Figure 6).

In the cases previously described in the literature, treatment was always laparotomic. In every case reported, laparotomy also had a diagnostic role when a certain diagnosis was not made through radiological examinations. Our case was the first laparoscopically treated.

## 5. Discussion

New onset ascites with acute abdomen in puerperium is a very rare condition and the differential diagnosis may be hard for the physician. Several causes of puerperal ascites have been described in the literature, all with a clinical picture similar to that of the spontaneous rupture of the bladder. One case of postpartum hepatic artery thrombosis has been described by Damman et al. in a patient presenting with fever, coma, ascites, ileus, jaundice and renal failure after delivery [18]. The impaired liver function suggested the hepatic origin of the clinical pictures. Gyang et al. reported the case of a missed diagnosis of acute postpartum pancreatitis in a patient showing abdominal pain, pyrexia, anemia and gross ascites a few days after instrumental delivery [19]. Bowel perforation is an uncommon complication that generally occurs during gestation and the most frequent locations are the rectum and sigma. This type of complication is generally linked to the presence of endometriosis infiltrating the intestinal wall. Acute pelvic and abdominal pain, diarrhea and fever are the typical symptoms [20]. Another cause of acute abdominal pain and abdominal effusion in the puerperium may also be the rupture of an ovarian cyst or ovarian torsion [21]. In these cases, a timely ultrasound approach may guide the diagnostic process.

Spontaneous rupture of urinary bladder (SRUB) following spontaneous vaginal delivery is an extremely rare condition and represents a surgical emergency. The few data in the literature do not help us to fully clarify the causes of this adverse event. It is usually described in association with recent trauma, malignant diseases, anatomical outflow obstructions, indwelling catheters, instrumentation, neurogenic bladder or a combination of these [3]. Despite the low incidence and non-specific symptomatology, diagnosis is often delayed and associated with a high mortality rate [22]. From our review, it emerged that the predominant clinical signs are abdominal pain and tenderness. Intraperitoneal fluid accumulation may cause intestinal and peritoneal irritations and urological symptoms, such as anuria [23]. Some authors reported cases characterized by signs of acute renal failure secondary to systemic absorption of urea and creatinine [24]. Serum chemistry abnormalities could be seen only after 24 h: creatinine, urea and potassium levels may be elevated, while sodium and chloride concentrations may be low [24,25,26]. Dysuria and hematuria were less frequently observed, which could mean that urological etiologies are misled, with delayed or missed diagnosis [27]. The history of urinary retention and the sudden relief or increase of pain, accompanied by small amounts of infected or blood-stained urine, is associated with a higher possibility of bladder rupture. In these cases, cystourethrography is regarded as the procedure of choice. Some authors suggested to use non-IV contrast CT in conjunction with retrograde cystourethrography, looking for intraperitoneal extravasation of contrast. This provides the benefit of cross-sectional images and the ability to distend the bladder in order to detect small perforations, avoiding contrast nephrotoxicity [12]. The aetiology of this condition is multifactorial. Urinary retention is not uncommon in the post-partum period. The epidural block proved to increase three times the risk of urinary retention. Other recognized risk factors are the use of systemic narcotics, perineal laceration, instrumental delivery and epidural analgesia during labor [12,27].

Another cause of bladder rupture is represented by the sustained pressure of the fetal head against the intraperitoneal portion of the bladder during forceful uterine contractions, provoking necrosis of the bladder dome. This is more likely if the patient is not catheterized, resulting in a distended bladder during labor. Other contributory factors include the presence of a pre-existing bladder diverticulum, prolonged second stage and high birth weight babies [12]. Not only may bladder damage occur during labor due to the lack of catheterization but also due to inadequate catheterization. Few cases of rupture after urinary catheterization without other predisposing factors have been described in the literature [28]. Four of these happened during labor vesical catheterization. Considering the morphology of the vesical lesion diagnosed in our and in other patients during-labor catheterization, this procedure may represent a risk factor for bladder rupture. When impromptu catheterization is performed inadequately, with thin catheters and excessive exertion of pressure, bladder damage may be caused [29]. In the final stages of labor, it is necessary to be careful during extemporaneous catheterization. Exerting an opposite force to that of uterine contractions and the fetal head can be responsible for the ischemic events in the bladder wall.

In our opinion, to prevent this kind of complication it would be better to use catheters of not too thin a gauge and avoid deep introduction of the catheter into the bladder in the final phase of labor.

Another risk factor evaluated is the fundal pressure during the second period of labor. In our case, no fundal pressure was applied. The role of this maneuver is understudied. The effects on the maternal perineum are inconclusive; fundal pressure emerged as an independent risk factor for anal sphincter tear at delivery and post-partum urinary retention [30,31,32]. There are no studies in the literature that correlate bladder rupture with this type of procedure.

There are some reports about concomitant bladder and uterine ruptures. We have excluded these cases from our systematic review, but with these types of complications, in addition to cystograms, some authors suggested that intravenous contrast-enhanced CT is at least as sensitive as cystography [33]. In fact, while considering nephrotoxicity of contrast-enhanced CT, the need to have an accurate diagnosis in a critically ill patient must be taken into account. Management differs between intra- and retroperitoneal rupture. Early diagnosis and prompt surgical treatment decreases the morbidity and mortality associated with this condition [5].

Retroperitoneal bladder rupture is commonly treated with bladder catheterization for 10 days. The classical treatment for intraperitoneal bladder rupture is surgical repair and urinary rest [28].

Muggia et al. managed this rare condition by percutaneous ascites drainage and long-lasting foley. However, the report described a case with small iatrogenic injuries. Large tears caused by blunt trauma clearly should be managed surgically [34].

Corriere et al. suggested strict criteria for conservative management of small intraperitoneal bladder perforation, including absence of infection, no bowel herniation, diagnosis within 12 h from the injury, no concurrent intrabdominal injuries and absence of ascites [26,35]. Operative treatment consists of urine removal from the peritoneal cavity, closing the rupture and instituting good vesical emptying [1,5]. From our review of the literature, it emerged that standard surgical intervention was laparotomic. To our knowledge, we report the first case of bladder rupture treated by a laparoscopic approach after normal vaginal delivery. An abdominal ultrasonography and a CT scan with contrast were performed and a diagnosis had been made. Laparoscopic management is indicated in hemodynamically stable women, when performed by surgeons expert in mini-invasive surgery. In our opinion, laparoscopy allows diagnosis and treatment of this complication. The superiority of laparoscopic approach over laparotomy in terms of lower blood loss, pain medication requirement, length of hospital stay and costs is well known [36,37]. This is important, considering the patient’s need of breastfeeding resumption. The young age of patients and the desire of fertility must be considered when surgical approaches are chosen. Laparotomic surgery could cause a higher incidence of periadnexal adhesions with deleterious effects on fertility, when compared to laparoscopy [37,38,39]. Furthermore, if ureteral stenting is required, in the suspicion of ureteral damage, particularly in the case of the contemporary rupture of the uterus and bladder, it can be performed under laparoscopic guidance without intraoperative fluoroscopy [40].

The strength of our study is the long period of time overviewed in the literature. We analyzed the cases of bladder rupture after normal vaginal delivery with no prior pelvic surgery or other risk factors that arose in the last 30 years. All the studies selected during the eligibility phase have been further evaluated by manual comparison of populations, study settings and authors to avoid overlapping cases. We also excluded from our review all cases in which patients had predisposing factors (such as cesarean section, previous pelvic surgery and contemporary uterine rupture) to minimize confounding factors and select bladder ruptures that occurred spontaneously after vaginal delivery.

The main limitation of this review is only case reports are included among the papers selected, this being due to the rarity of this complication.

## 6. Conclusions

Gynecologists must be aware of bladder rupture after spontaneous delivery—a rare but insidious occurrence. Abdominal pain and blood tests indicating kidney failure should suggest the presence of this complication. On the basis of the results of our review, we suggest a diagnostic algorithm (Figure 6). Firstly, an abdominal ultrasound evaluation should be performed. In case of an inconclusive ultrasound result, a patient still hemodynamically stable and with abdominal free fluid should undergo a computerized tomography. If this doesn’t enable a decisive diagnosis, cystourethrography is the gold standard to identify bladder perforation. The therapeutic choice depends on the type of bladder rupture. Retroperitoneal rupture is commonly treated with bladder catheterization. The classical management for intraperitoneal rupture of the bladder is surgical repair and urinary rest. In the literature, all cases have been treated by laparotomic approach. However, laparoscopy is a possible and effective alternative as a diagnostic tool, the performance of which must take into consideration the patient’s condition and the operators’ experience. A quick diagnosis and an adequate surgical approach are crucial for the resolution of this rare complication.

## Figures and Tables

**Table 1:** JBI Critical Appraisal Checklist for Case Reports.

Title	Author, Year	D1	D2	D3	D4	D5	D6	D7	D8
Spontaneous bladder rupture after normal vaginal delivery: a postpartum emergency [8]	Roberts C et al., 1996	Yes	Yes	Yes	Yes	Yes	Yes	Yes	Yes
Spontaneous rupture of bladder in puerperium [3]	Wandabwa J et al., 2004	Yes	Yes	Yes	Yes	Yes	Yes	Yes	Yes
Asymptomatic bladder rupture in a primigravida: late manifestation and delayed diagnosis [9]	Puri M et al., 2005	No	Yes	Yes	Yes	Unclear	Unclear	Yes	Unclear
Spontaneous rupture of urinary bladder in puerperium [10]	Pal DK et al., 2005	Yes	Yes	Yes	Yes	Yes	Yes	Yes	Yes
Bladder rupture caused by postpartum urinary retention [11]	Dueñas-García OF et al., 2008	Unclear	Yes	Yes	Yes	Yes	Yes	Yes	Yes
Two cases of intraperitoneal bladder rupture following vaginal delivery [12]	Png KS et al., 2008	Yes	Yes	Yes	Yes	Unclear	Yes	Yes	Yes
Spontaneous puerperal extraperitoneal bladder wall rupture in young woman with diagnostic dilemma [13]	Sabat D et al., 2015	Yes	Yes	Yes	Yes	Yes	Yes	Yes	Yes
A late presentation of spontaneous bladder rupture during labor [1]	Farahzadi A et al., 2016	Yes	Yes	Yes	Yes	Unclear	Yes	Yes	Yes
Acute abdomen syndrome due to spontaneous intraperitoneal bladder rupture following vaginal delivery [14]	Habek D et al., 2017	Yes	Yes	Yes	Yes	Yes	Yes	Yes	Yes
Delayed diagnosis of spontaneous bladder rupture: a rare case report [15]	Qiao D et al., 2018	Yes	Yes	Yes	Yes	Yes	Yes	Yes	Yes
Spontaneous rupture of bladder in a primipara [16]	Ekuma-Nkama EN et al., 2019	Yes	Yes	Yes	Yes	Yes	Yes	Yes	Yes
Surgical management of spontaneous post-partum bladder rupture in an Amazonian emergency hospital [17]	Marcos da Silva Barroso F et al., 2020	Yes	Yes	Yes	Yes	Yes	Yes	Yes	Yes
Missed bladder rupture following vaginal delivery: Possible role of assessing ascitic fluid creatinine levels? [2]	Hadian B et al., 2020	Yes	Yes	Yes	Yes	Unclear	Yes	Yes	Yes

**Table 2:** Cases in the literature.

Title	Author, Year	Age	Time to Rupture (Days)	Baby Weight (g)	Parity (Postpartum)	Type of Delivery	Previous Surgery	Catheterization	Catheterization during Labor	Symptoms	Diagnosis	Treatment
Spontaneous bladder rupture after normal vaginal delivery: a postpartum emergency [8]	Roberts C et al., 1996	29	3	3400	2	VD	No	Yes	NA	Increasing abdominal pain	XR + catheterization + LPT	LPT
Spontaneous rupture of bladder in puerperium [3]	Wandabwa J et al., 2004	20	9	2800	1	VD	NA	Yes	NA	Severe lower abdominal pain, abdominal distension, fever, difficulty in breathing	US + LPT	LPT
Asymptomatic bladder rupture in a primigravida: late manifestation and delayed diagnosis [9]	Puri M et al., 2005	NA	4	NA	NA	VD	NA	NA	NA	Urinary Retention, abdominal distension	XR + cystogram + LPT	LPT
Spontaneous rupture of urinary bladder in puerperium [10]	Pal DK et al., 2005	23	1	NA	1	VD	None	Yes	NA	Distended abdomen, vomiting and ol- iguria	CT + cystography + LPT	LPT
Bladder rupture caused by postpartum urinary retention [11]	Dueñas-García OF et al., 2008	NA	3	NA	1	VD	NA	Yes	NA	Abdominal pain, oliguria, hematuria	XR + US + LPT	LPT
Two cases of intraperitoneal bladder rupture following vaginal delivery [12]	Png KS et al., 2008	34	2	2685	1	VD	NA	Yes	NA	Abdominal distension, acute renal failure	XR + CT + LPT	LPT
Spontaneous puerperal extraperitoneal bladder wall rupture in young woman with diagnostic dilemma [13]	Sabat D et al., 2015	20	6	2830	NA	VD	NA	Yes	Yes	Abdominal pain and distension, oliguria	Paracentesis, US, CT, LPT	LPT
A late presentation of spontaneous bladder rupture during labor [1]	Farahzadi A et al., 2016	25	20	NA	1	VD	NA	Yes	Yes	Abdominal pain and distension	US + LPT	LPT
Acute abdomen syndrome due to spontaneous intraperitoneal bladder rupture following vaginal delivery [14]	Habek D et al., 2017	28	4	3350	1	VD	NA	Yes	Yes	Acute abdomen syndrome, diarrhea and oedema	US + CT + LPT	LPT
Delayed diagnosis of spontaneous bladder rupture: a rare case report [15]	Qiao D et al., 2018	23	5	3600	2	VD	NA	Yes	NA	Fever, oliguria, massive ascites	US, MRI, CT, cystoradiography and cystoscopy, LPT	LPT
Spontaneous rupture of bladder in a primipara [16]	Ekuma-Nkama EN et al., 2019	30	3	3254	1	VD	NA	Yes	Yes	Severe abdominal pain, oliguria and hematuria	XR + US + LPT	LPT
Surgical management of spontaneous post-partum bladder rupture in an Amazonian emergency hospital [17]	Marcos da Silva Barroso F et al., 2020	30	3	3200	2	VD	No	NA	NA	Sudden abdominal pain, vomiting	US + CT + LPT	LPT
Missed bladder rupture following vaginal delivery: Possible role of assessing ascitic fluid creatinine levels? [2]	Hadian B et al., 2020	27	3	NA	1	VD	No	NA	NA	Drowsiness and abdominal distention	CT + LPT	LPT

## Data Availability

The authors confirm that the data supporting the findings of this study are available within the article.

## Appendix A

**Table A1:** JBI Critical Appraisal Checklist for Case Reports.

D1.	Were patient’s demographic characteristics clearly described?
D2.	Was the patient’s history clearly described and presented as a timeline?
D3.	Was the current clinical condition of the patient on presentation clearly described?
D4.	Were diagnostic tests or assessment methods and the results clearly described?
D5.	Was the intervention(s) or treatment procedure(s) clearly described?
D6.	Was the post-intervention clinical condition clearly described?
D7.	Were adverse events (harms) or unanticipated events identified and described?
D8.	Does the case report provide takeaway lessons?
